# The Control-Experiment and the Enthusiasts

**Published:** 1911-09

**Authors:** 


### The Control-Experiment and the Enthusiasts

We called the attention of our readers in our last issue to the fact that the opening of the new year would be the occasion of the demonstration to all concerned of a larger life, of a more vigorous, virile and up-to-date activity on the part of the journal, and to promote this end their coöperation was earnestly solicited.

We also think our new year not an unsuitable time for a brief survey of some of the more interesting and potential events of recent years—therefore our subject, The Control-Experiment and the Enthusiasts.

Students of the history of medicine are constantly and continuously confronted with the evidence that progress in medicine, like progress in science in general, owes an enormous obligation to the men who differed chiefly from their fellows in their larger enthusiasm. Some of these men had more knowledge—all had more enthusiasm. We believe that the rapid progress of the last thirty years, since Koch introduced the control-experiment expressed in his law of pathogenesis as compared with the progress of the century or even the centuries previous, is in great part due to the fact not that the earlier times were wanting in earnest enthusiastic workers and investigators, but that the results of their observations were too frequently misleading from the fact that the accepted proofs of those results had not been scrutinised and tested by the accurate control-experiments now in constant use over the whole world of medicine.

To recall the slow growth of the knowledge as to the cause of disease and with greater particularity of the knowledge of the role of the pathogenic germs in causing disease will make our meaning clear. Marcus Antonius Plenciz (1762) gave a clear statement of his belief in the germ theory of disease. Spallanzani, (1769) ; Schulze, (1836) ; Schwann, (1839) and Henle, (1840-1853), contributed most important observations towards establishing the facts included in the theory, which facts by the way had been demonstrated by Bassi, (1837) ; in the case of the fatal malady of the silk worm—Pèbrine.

However, neither the work of these illustrious men nor that of their even more distinguished successors of the next decade, Dovaine, Pollender, Rindfleisch, Billroth, Lister and Pasteur, was so potential in ultimate results, so transforming in many directions as the work of Koch (1881).

Koch taught the world how to demonstrate the facts of the pathogenesis of the germ diseases as propounded by Plenciz more than a century and a quarter before, and progress in this important field of medicine waited all that long interval for the guide, the compass—the control-experiment.

How essential and all important in research is a right method we may possibly get a notion by a moment's survey of a couple of instances in which earnestness, sincerity and lofty purpose were present in a marked degree, where observations were made and results proclaimed in all sincerity of faith and conviction and yet in which future demonstrations proved or will prove that the observations and the results were unreliable, misleading for want of accurate scrutiny and definite testing by suitable control experiments. We refer to the modern systems of medicine—Homeopathy and Quimby-Eddyism.

The founder of homeopathy, an honest and well equipped member of the medical profession, announced about 1810 his so thought discovery as to the nature and cause of disease and as to the way in which medicines were curative. As proclaimed by him the cause of disease was never material but always immaterial, dynamic, spiritual; and medicines were curative in direct proportion as they were diluted, attenuated, raised in potency as they were reduced in weight.

We believe it may be said in historic accuracy and fairness that this system of medicine was largely speculative in its conception and promotion; that it rose and flourished with distinct disregard to the now well known scientific principle that discoveries must be thoroughly tested by control-experiments before they are accepted by students of the subject.

However the years passed, men grew, instruments of precision came, students inquired of nature and were taught by it, the belief that certain diseases were caused by certain micro-organisms ceased to be a belief because it became a demonstrated fact of scientific knowledge.

Every discovery of a pathogenic germ in plants, in animals or in man—and there are hundreds of such—was a demonstration of the material nature of the cause of disease in direct opposition to the faith of homeopathy.

The control-experiment has done its work in this particular direction so far as the cause of disease is concerned, and is rapidly doing its work in like manner in therapeutics.

The rise of the system of medicine, Quimby-Eddyism, presents many features quite analagous to those of homeopathy, at least there is in both a generous enthusiasm, a complete reliance upon the supposition that with good motives, good intentions, good purposes, methods will, of course, be all that may be desired. The former taught that the cause of diseases was not material, but spiritual; the latter teaches that there is no such thing as pain or disease, but that those who are in error think there is, and so thinking make a mistake. Homeopathy taught that disease was best cured by highly attenuated, spiritualised remedies. Quimby-Eddyism teaches that the mind, our most spiritual part, is the only cause and the only cure of disease.

It would seem that as the control-experiment gradually but persistently invaded the realm of speculation in homeopathy transforming the enthusiasm of belief into the reality of knowledge so we may reasonably expect the same system some day to enter the realm of Quimby-Eddyism with the demonstrations of the reality of matter, of air and water, of bread and meat, of thirst and hunger, of pain and of disease; even possibly the proof of demonstration that prevention of disease may be more satisfactory than its cure, and that in the question of curing disease, some treatments are better than others. We believe the control-experiment bears an important message for the enthusiast.

